# Species Matter: Wood Density Influences Tropical Forest Biomass at Multiple Scales

**DOI:** 10.1007/s10712-019-09540-0

**Published:** 2019-06-03

**Authors:** Oliver L. Phillips, Martin J. P. Sullivan, Tim R. Baker, Abel Monteagudo Mendoza, Percy Núñez Vargas, Rodolfo Vásquez

**Affiliations:** 1 School of Geography, University of Leeds, Leeds, LS2 9JT UK; 2 Jardín Botánico de Missouri, Jr. Bolognesi, 19230 Oxapampa, Peru; 3 Universidad de San Antonio Abad del Cusco, Av. de La Cultura 773, 08000 Cuzco, Peru

**Keywords:** Amazon, Tropical forests, Species, Identity, Carbon, Biomass, Dynamics

## Abstract

The mass of carbon contained in trees is governed by the volume and density of their wood. This represents a challenge to most remote sensing technologies, which typically detect surface structure and parameters related to wood volume but not to its density. Since wood density is largely determined by taxonomic identity this challenge is greatest in tropical forests where there are tens of thousands of tree species. Here, using pan-tropical literature and new analyses in Amazonia with plots with reliable identifications we assess the impact that species-related variation in wood density has on biomass estimates of mature tropical forests. We find impacts of species on forest biomass due to wood density at all scales from the individual tree up to the whole biome: variation in tree species composition regulates how much carbon forests can store. Even local differences in composition can cause variation in forest biomass and carbon density of 20% between subtly different local forest types, while additional large-scale floristic variation leads to variation in mean wood density of 10–30% across Amazonia and the tropics. Further, because species composition varies at all scales and even vertically within a stand, our analysis shows that bias and uncertainty always result if individual identity is ignored. Since sufficient inventory-based evidence based on botanical identification now exists to show that species composition matters biome-wide for biomass, we here assemble and provide mean basal-area-weighted wood density values for different forests across the lowand tropical biome. These range widely, from 0.467 to 0.728 g cm^−3^ with a pan-tropical mean of 0.619 g cm^−3^. Our analysis shows that mapping tropical ecosystem carbon always benefits from locally validated measurement of tree-by-tree botanical identity combined with tree-by-tree measurement of dimensions. Therefore whenever possible, efforts to map and monitor tropical forest carbon using remote sensing techniques should be combined with tree-level measurement of species identity by botanists working in inventory plots.

**Electronic supplementary material:**

The online version of this article (10.1007/s10712-019-09540-0) contains supplementary material, which is available to authorized users.

## Introduction

Tropical forests contain more species and biomass than any other biome on Earth. While they are being rapidly degraded and deforested, large areas of relatively intact tropical forest still exist, particularly in the Amazon and Congo basins. Wherever they persist, tropical forests contribute hugely to societies, economies, and human well-being, providing vital services that sustain people and nations (Watson et al. 2018). For example, dozens of the tree species in South American forests are also cultivated or domesticated, and hundreds more are close relatives (Levis et al. 2017). Meanwhile, the carbon sink into mature forests has mitigated deforestation and fossil fuel emissions in many Amazon nations for decades (Phillips and Brienen 2017; Phillips 2018; Vicuña Miñano et al. 2018), so slowing the rate of climate change. These services are all under threat, however, with climate change itself a leading concern. Tropical lands have been warming fast, and continued warming is projected to combine with stronger droughts and potentially lead to crossing ecological thresholds (e.g., Good et al. 2018), bringing increased risks to biomass storage, tree species, and human societies.

This unique nexus of values and threats in tropical forests means that measuring and mapping their biophysical properties—and then tracking changes—are central goals of global environmental science. Yet because of their extent and complexity, tropical forests are challenging to measure and monitor with precision. For the key property of biomass—from which we may derive carbon storage per unit area—space-borne and airborne sensor technologies are increasingly used to infer biomass [Zolkos et al. 2013; Minh et al. 2014; Coomes et al. 2017; Jucker et al. 2018a; Duncanson et al. (this volume)]. Laser scanning enables precise measurement of canopy height, and if done at sufficient intensity can reveal the three-dimensional structure of trees, while space-borne radar offers potentially global-scale assessment of forest structure. Optical sensing of canopies is widely used to infer vegetation state, such as distinguishing forest from non-forest. However, a key technical limitation is that no technology directly measures a critical determinant of every tree’s biomass—its identity. This represents a fundamental challenge, especially given that the single most remarkable and celebrated feature of tropical forests is their extraordinary diversity of species and variation in tree composition (e.g., ter Steege et al. 2013). Indeed, tropical tree species composition varies at all scales from a few metres to across the whole biome due to factors that include climate, geomorphology, nutrient supply, evolutionary history, and anthropogenic impacts (e.g., Salo et al. 1986; Gentry 1988; Tuomisto et al. 1995; Condit et al. 2002; ter Steege et al. 2006; Honorio Coronado et al. 2009; Asner et al. 2017; Levis et al. 2017).

Accurate measurement of most tropical trees’ biological identity requires direct observation from the ground, supported by collection and subsequent careful identification of the herbarium vouchers by trained botanists (Baker et al. 2017). Since biological composition determines the physical composition of forests in terms of leaf (e.g., Fyllas et al. 2009; Asner et al. 2017) and wood properties (Muller-Landau 2004; Patiño et al. 2009; Baraloto et al. 2011), then an inability to perceive biodiversity may significantly hinder estimation of biomass and carbon storage. Yet how much the variation in tropical forest species actually matters for biomass mapping remains controversial. The aims of this paper are to explore and quantify this issue for tropical forests and then suggest how the difficulties faced by current remote Earth Observation techniques in mapping tropical forest species compositional variation and biomass density might be mitigated. By combining literature and new analysis, we examine the issue from the scale of individual tropical tree up to whole continents and assess its impact on Amazon biomass estimates.

### In Practice, Does Diversity Matter?

Forests are made mostly of trees, and in tropical forests these come in extraordinary variety. There can be 300 tree species in a 100-by-100-m patch of Amazon forest. Remarkably, these single-hectare tropical samples contain more woody plant species than are found in all of Earth’s boreal forests—an area some nine orders of magnitude greater. Tropical Peru has almost 5000 tree species recorded, with new species being discovered every year (Vásquez et al. 2018), while the temperate UK has less than 50. In Amazonia, there are as many as 16,000 tree species (ter Steege et al. 2013). With huge floristic diversity, it is reasonable to expect a high degree of functional diversity too, including in the key attributes that affect tree biomass (Baker et al. 2009; Baker 2018). There is ongoing debate as to whether diversity helps support higher biomass, and if so how (e.g., Bunker et al. 2005; Sullivan et al. 2017), but here we are interested in the question of how *differences* in the composition of species from one diverse forest to another impact biomass. Thus, here it is the different taxonomic and evolutionary identities of the tree species present which are hypothesised to matter, not the number of species per se.

While the biomass contained by any individual tree is determined by many factors, these are reducible to just two: (1) its size—the volume of wood—and (2) the amount of matter per unit volume or its density. (Here we use the standard definition of ‘basic specific gravity’, defined as the ratio of the oven-dry mass of a wood sample divided by its green volume, e.g., Chave et al. 2006.) The genetic identity of a tree affects both how big it can become *and* how dense it is (Baker et al. 2004; Fauset et al. 2015; Coelho de Souza et al. 2016). Various studies have shown that these effects are largely independent (e.g., Turner 2001; Coelho de Souza et al. 2016; Hietz et al. 2017): across tropical tree species, maximum size (height, diameter, volume) and density of wood are largely uncorrelated. Since size is a poor predictor of wood density it follows we cannot use the dimensions of trees to infer their density. The fundamental disconnect between size and wood density means that measuring size alone can never capture all information needed to derive biomass.

**Fig. 1:**
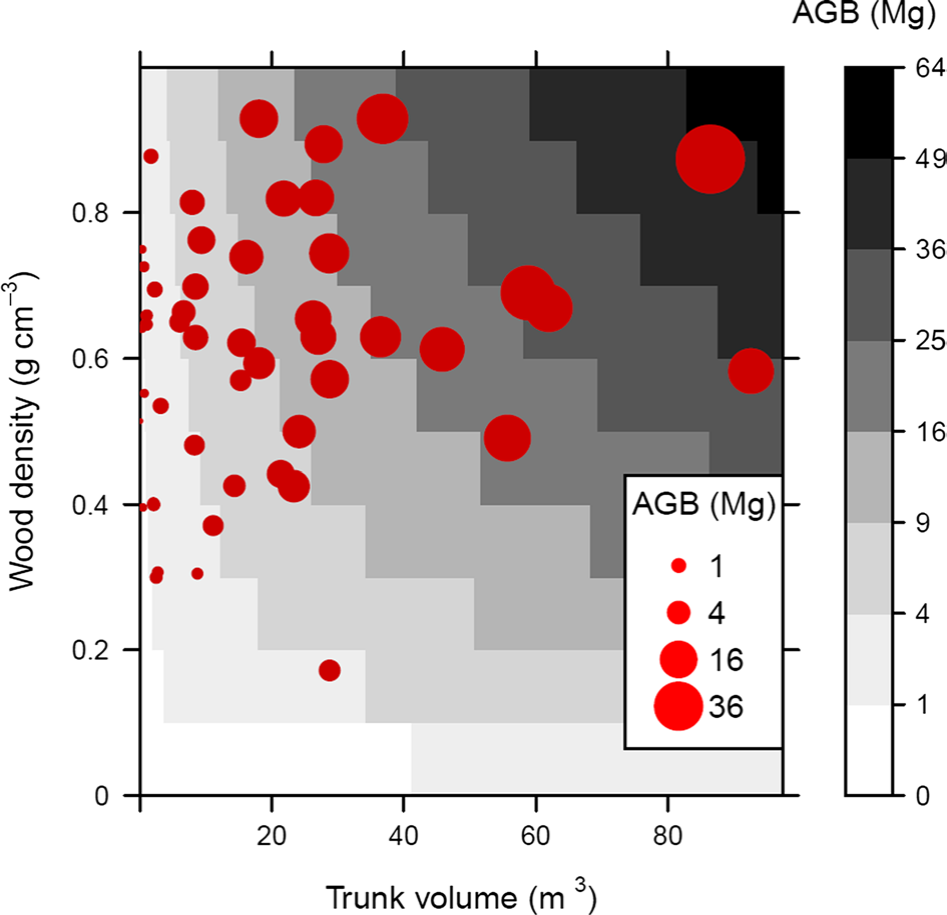
Direct measurement of tropical trees shows that wood density and size each independently control biomass. Red points represent 51 forest trees destructively sampled and weighed by Goodman et al. (2014a, b) in Amazonian Peru. Point areas are proportional to the *actual, directly measured* aboveground biomass (AGB) of each tree, plotted against their trunk volume and directly measured wood density. Trunk volume was estimated as basal area multiplied by tree height. The greyscale background depicts a quasi-continuous allometric estimate of AGB for combinations of tree volume and wood density. To do this, the Chave et al. (2014) allometric equation was solved for each combination of diameter and wood density, with tree height estimated using a three-parameter Weibull model fitted to all trees in the Goodman et al. (2014a, b) dataset

Consequently, in species-diverse tropical forests tree biomass varies greatly even for a fixed tree size. In southern Peru, Goodman et al. (2012, 2014a) identified, harvested, and painstakingly weighed 51 individual trees as large as 169 cm diameter. We plot these data here to illustrate how volume and wood density combine to determine biomass (Fig. 1). Canopy trees with similar dimensions have very different biomass. For example, a *Cavanillesia umbellata* canopy tree with wood density measured at 0.132 g cm^−3^ had a dry aboveground mass of 2.3 Mg, while an *Apuleia leiocarpa* individual with wood density of 0.855 g cm^−3^ weighed 12.2 Mg, in spite of having slightly *less* wood volume. Further, in multivariate allometric models of tree biomass based on harvested tropical trees, wood density is the most important factor after stem diameter in explaining tree biomass—entering models *before* height (e.g., Chave et al. 2014; Goodman et al. 2014a)—with biomass scaling almost linearly with wood density (Chave et al. 2014). With the huge range in wood density of species present locally, it is essential to know identity in order to estimate tropical tree biomass with confidence.

Yet it does not necessarily follow that the impact of identity on biomass will persist at the larger scales of interest to most Earth Observation questions. Here, we seek to address the question of the extent to which species composition impacts on forest biomass at stand, landscape, and larger scales by reviewing current evidence and developing new analyses. Ultimately, we wish to shed light on the pervasiveness of species effects, focussing on South American tropical forests due to the relatively larger literature here.

At the very largest scales, some spatial variation in forest biomass driven by wood density is already recognised. For example in the neotropics, dry forest trees generally have greater wood density (Chave et al. 2006), and it has long been known that successional forests have lower wood density than mature forests (e.g., Brown and Lugo 1990). However, across the tropical moist forest biome we could not find any analysis at the pan-tropical level as to whether wood density varies systematically continent to continent.

At the sub-continental scale, concerted, species-driven differences in wood density prevail even within the same biome and same successional stage. In mature African moist forests, soil-related compositional differences cause significant differences in basal-area-weighted wood density, with forests on relatively fertile acrisols and cambisols having 10% lower values (0.609 and 0.617 g cm^−3^) than on arenosols (0.660 g cm^−3^) and 20% lower than swamp forests on histosols (0.728 g cm^−3^) (Lewis et al. 2013). Basal-area-weighted wood density is also significantly higher for Central African forests than their West or East African counterparts (Lewis et al. 2013). Likewise, South American forests differ greatly when comparing central with western Amazonia, with 16% lower per-stem wood density in the west caused by differences in floristic composition (Baker et al. 2004). This is in spite of similar climate and instead is linked to differences in stem turnover rates, with the western forests much more dynamic (Phillips et al. 2004), often associated with more cation-rich and structurally poor soils (Quesada et al. 2012): trees grow and die faster here and this favours species which are adapted to exploiting gaps quickly. While different life-history strategies are found in *all* Amazon forests, the mean wood density in the slow turnover forests on the Guiana Shield is 50% greater than in the fast turnover forests in south-west Amazonia (ter Steege et al. 2006), helping drive much greater standing biomass in the north-east (Malhi et al. 2006; Johnson et al. 2016). This large-scale species-driven difference in biomass is invisible in space-borne LiDAR-derived biomass estimates (c.f. Mitchard et al. 2014), but is accounted for in hybrid biomass maps that attempt to combine plot-derived measures of species differences across space with LiDAR measurements (Avitabile et al. 2016).

While the broad difference between north-east and south-west Amazon forests is clear, uncertainties remain, including the exact nature of the relationships between AGB and wood density, and between mortality rates and wood density, and crucially whether these relationships also persist at smaller geographical units. Some evidence suggests that at finer scales a more nuanced situation prevails. Within western Amazonia, Landsat-based analyses have revealed great variation in spectral types of forest, starting with the seminal study of Salo et al. (1986). In combination with fieldwork, spectral variation has been linked to variation in species and subtle geomorphological, edaphic, and geological controlling factors have been revealed (e.g., Tuomisto et al. 1995; Higgins et al. 2011). Recently, using airborne hyperspectral sensing variation in canopy function has been explored here at high resolution (Asner et al. 2017; Draper et al. 2019). These analyses all confirm both discrete and continuous variations in canopy function across Peru’s forests.

Recent investigations also show how soil and species differences affect tropical forest wood density in regional and landscape scales. In the Central African Republic, Gourlet-Fleury et al. (2011) found differences in wood density of 20% between forest types in one landscape, mediated by soil nutrients and drainage. Similarly, in north-west Amazonia and in French Guiana, Baraloto et al. (2011) found that forests with richer soils tended to support trees with lower wood density. In particular, they report highest wood density in white sand forests, with these having nearly 20% higher average wood density than terra firme and seasonally flooded forests. In Borneo, Jucker et al. (2018a, b) also found large, landscape-level differences in community-mean wood density. As in Amazonia and French Guiana, in Borneo it is white sand forests that have highest wood density. Jucker’s (2018a) analysis also reveals smaller but significant variation in basal-area-weighted wood density between and within different sites in north-east Borneo, including additional substrate-related variation between forests growing on alluvial versus sandstone-derived soils. Here, forest-wide wood density is, respectively, 10% lower and 10% greater than the Borneo-wide forest mean (Qie et al. 2017). Therefore, in every case—across African, South American, and Asian landscapes—failing to account for habitat-related variation in wood density significantly biases AGB estimates. In Borneo, where extensive field sampling was combined with hyperspectral imaging, wood density estimates inferred from canopy leaf spectra vary with topography by as much as 40% at the 1-ha scale (Jucker et al. 2018b). In sum, complex variation in biodiversity across tropical forest landscapes is the rule, not the exception, and this matters for biomass mapping.

While quantifying how floristic variation impacts AGB is critical for mapping purposes, the question of *why* tropical forests’ wood density varies spatially is equally important. In the white sand case, Borneo and South America are biogeographically isolated from one another so the consistent response implies independent convergence in function driven by selective pressures, possibly as a result of low nutrient availability favouring more conservative, slow-growing species. Topographic differences in wind disturbance (Fortunel et al. 2014) and drought stress (Cosme et al. 2017) may also control local-scale wood density variation. In Amazonia, the large regional differences in wood density are related to greater dominance by light-wooded families in the south-west (e.g., palms and mimosoid legumes) and dense-wooded families in the north-east (e.g., Sapotaceae and caesalpinoid legumes). This may be ultimately driven by the unique biogeographic history of the Guiana Shield and the edaphic differences between deep, weathered soils in east-central Amazonia and less developed soils in the Andean forelands affecting forest dynamics (Fyllas et al. 2009; Baraloto et al. 2011; Quesada et al. 2012; Johnson et al. 2016). Forest structure and dynamics are not only causally linked, but impact other carbon pools too. For example, wood density also affects carbon storage after death: light-wooded forests store less necromass than dense-wooded systems (Chao et al. 2009). The mechanistic links between environment, structure, composition and dynamics are relevant for the practical task of remote sensing of biomass as they point to forest properties measureable remotely which may be used to infer composition and hence wood density.

In sum, species variation impacts AGB at landscape, regional and continental scales. Yet given the scale of the biome we have only begun to evaluate how biodiversity affects mature forest biomass and wood density. Across the 6 million km^2^ extent of Amazonia, we lack case study analysis of impacts on AGB, especially at fine scales and in larger, biogeographic units. Here we aim to address these gaps. We first analyse plot-based inventories from one of the best-sampled Amazon landscapes, backed up by some of the most comprehensive botanical work anywhere in the tropics. Secondly, using the latest published data from the RAINFOR plot network we revisit the question of how much wood density matters for large-scale Amazon-wide forest biomass and forest dynamics, and to what extent these relationships hold in geoecological units within Amazonia. Finally, we combine the new and recent ecological work reporting wood density to document for the first time *basal*-*area-weighted forest wood density* estimates at multiple scales across the tropics.

## Methods

**Fig. 2:**
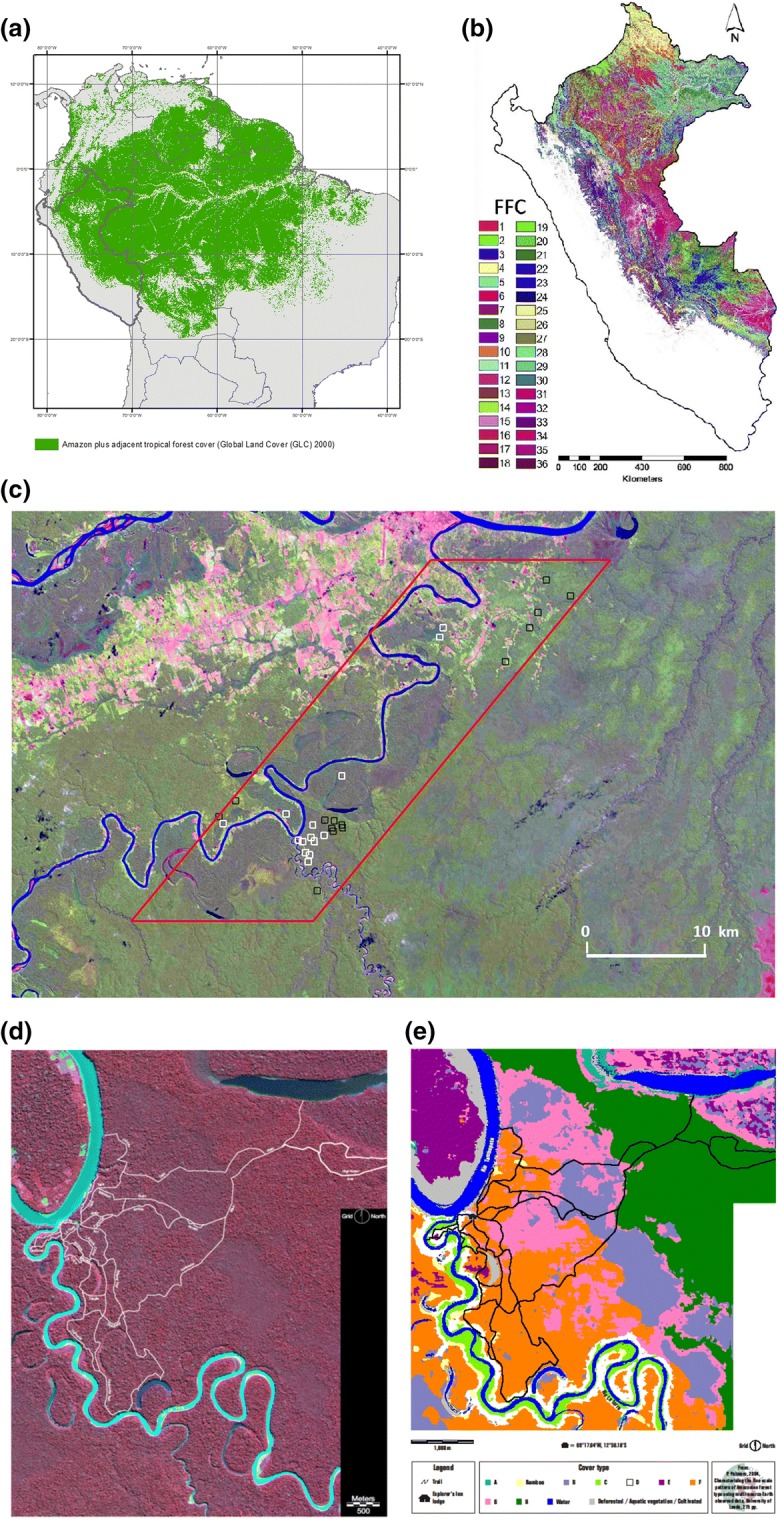
Multiple perspectives on Amazon forest diversity. The figure depicts the study region and forest-type variation sensed with imagery acquired contemporaneously with the floristic and ecological inventories. **a** Top left. *South American forest cover* in the year 2000 and location of Peru. **b** Top right. *Western Amazon forest ‘Functional Classes’* inferred from hyperspectral imagery by Asner et al. (2017) in Peru, with location of the lower Tambopata region in south-east Peru highlighted in red box. **c** Centre. *Our sample landscape* outlined as 15-by-40-km zone oriented along the lower Tambopata river. Young or disturbed vegetation regenerating after fluvial and anthropogenic clearing represents ≈ 10% of the landscape and was not sampled. Black icons represent locations of floristic sample plots in ‘Altura’ forest (Pleistocene sediments); red icons sample plots in ‘Bajio’ forest (Holocene sediments). In this false-colour image, the purple-green hued vegetation closer to the river corresponds to ‘Bajio’; the brighter green away from the river is ‘Altura’. Landsat imagery from https://landsat.usgs.gov/landsatlook-images, level-1 data product using imagery from 1999 to 2001, centred on Landsat path 114 row 175 and treated with a three-standard-deviation stretch. **d** Below left. *The best-sampled forests* centred on Tambopata reserved zone. Note the fine-scale variation in canopy composition and structure driven by small elevational differences. The total elevational range within this IKONOS image is ≈ 30 m. **e** Below right: *Ground-truthed interpretation of IKONOS imagery based on direct observation of geomorphology, hydrology and vegetation species and structure*. Colours correspond to ten distinct local forest types (Gentry 1988, Conservation International and Foster 1994): among-habitat diversity in species composition and associated functional traits is greater than the basic Altura–Bajio dichotomy. ‘Altura’ forest is dark green here (ancient Pleistocene river terrace); ‘Bajio’ forest includes orange and pink (different levels of Holocene terraces) as well as swamp and fluvial successional systems. Images from Palmero (2004)

For our landscape-scale evaluation of species impacts on biomass, we focus on the lower Tambopata region, in south-eastern Peru. Thirty-five years of botanical collecting have generated a relatively complete knowledge of the flora of the region, and forest inventory and monitoring provide sample plots in intact and human-modified forests, including 1-ha permanent plots and 0.1-ha inventories using modified Gentry transects (Gentry 1988; Clinebell et al. 1995; Phillips and Miller 2002; Phillips et al. 2003; Pallqui et al. 2014). Variation in fluvial disturbance, soil chemistry, and land use all affect tree species and human livelihoods here (e.g., Phillips et al. 2003; Lawrence et al. 2005). The landscape is divisible into two major landforms reflecting areas with recent Holocene deposition (‘depositional’) and more weathered Pleistocene sediments now being eroded (‘erosional’) (Kalliola et al. 1992; Räsänen et al. 1992; Osher and Buol 1998). This reflects the folk categories recognised by local dwellers, ‘Altura’ and ‘Bajio’, who account for subtle differences in elevation and forest resources (Phillips et al. 2006). Classifications derived from larger-scale maps of the Peruvian Amazon (e.g., Asner et al. 2017; Peru Ministerio de Ambiente 2015) are consistent with local perceptions of the natural forest environment but don’t fully coincide. We therefore use here the local terms ‘Altura’ and ‘Bajio’ and the locally equivalent ‘Pleistocene’ and ‘Holocene’ terminology. We centre our analysis on the mature-forest landscape in a roughly 600-km^2^ region of the lower Tambopata (Fig. 2), an area with more than 1000 tree species (Phillips et al. 2003). We only use mature-forest plots that are botanically identified as our interest is to identify floristic variation that is geomorphologically associated, rather than due to land-use change. Sampling was conducted between 1983 and 2007 (median date 1998) and stratified collaboratively with local residents by geomorphology using Landsat imagery, with exact locations randomised within target habitats. While the landscape has a fluvial signature, to our knowledge the samples included here have not been subject to recent river flooding.

Following established procedures (e.g., Baker et al. 2004; Lewis et al. 2013; Malhi et al. 2014), we derive taxon-specific wood density (WD) from a large-scale Global Wood Density database (Zanne et al. 2009) and estimate AGB at the tree and plot levels (Table S1). We use standard methods to estimate biomass (Chave et al. 2014) accounting for stem diameter, tree species identity, and height derived from forest-type-specific height-diameter allometries (Sullivan et al. 2018). We also accounted for palm-specific allometry (Goodman et al. 2014b) and implemented these procedures in the BiomasaFP R package (Lopez-Gonzalez et al. 2015).

Because we wanted to assess the impact of using incomplete biological information (‘identity-poor’) on forest biomass estimates, we first allocated the best available WD per stem and then calculated alternative averages at different scales, using these mean values instead of the best available values to test questions about *how spatial scale of identity impacts WD*. Thus wood density is allocated to individual trees *optimally* by accounting for actual tree-by-tree identity (to generate *community wood density*), and then *instead* by applying to each tree just: (2) the *plot-mean WD*; (3) the *forest*-*type mean WD*; (4) the Tambopata *landscape-mean WD*; and (5) the *Amazon*-*wide mean* WD values. Thus, for example, (2) represents a situation where we have perfect knowledge of plot average wood density but no knowledge of individual tree identity, (3) represents a situation where we have knowledge only of forest-type mean wood density, and so on. In each case, we compute mean values using simple abundance-weighting and by weighting by basal area of each species. The different procedures to compute forest wood density are summarised in Table 1. We use our AGB estimates to quantitatively address three linked questions for our study landscape: (1) To what extent does wood density vary among Pleistocene and Holocene landscapes at the tree level and the plot level?; (2) Does accounting for wood density change the expected relationship between forest basal area and biomass?; and (3) What are the consequences in terms of bias and uncertainty of using different ‘identity-free’ estimators of aboveground biomass?

**Table 1:** Terms used to report tropical forest wood density (g cm^−3^) in this paper, together with their definitions and data requirements. *Identity*-*rich metrics* use species identity to derive the wood density of each and every tree. *Identity*-*poor metrics* simply apply aggregate mean values to all trees, plots, forest types, or landscapes. We include these latter approaches which implicitly assume that species identity does not matter to assess the impact of using incomplete biological identities on forest biomass estimates. Note that whether identity-poor or identity-rich, the community-mean, plot-mean, forest-type-mean, landscape-mean, Amazon-mean wood density metrics can all be either abundance-weighted or basal-area-weighted. We recommend use of *identity*-*rich basal*-*area-weighted wood density* whenever possible (highlighted here and Table 2)

Term	Definition	Data required to estimate
***Identity-rich metrics***		
Species wood density	Species ‘basic specific gravity’, the oven-dry mass of a wood sample divided by its green volume (cf. Chave et al. 2006)	Ideally based on multiple individuals and accounting for radial variation from core to pith. Either from compilations (Zanne et al. 2009) or local measurements (e.g., Goodman et al. 2014a, b)
		If no species wood density measurements available, allocate the genus-level mean, else the family-level mean (Baker et al. 2004)
Community-mean wood density	Community-mean wood density (WD), based on each tree’s species wood density weighted by the abundance of each species	Additionally requires species-abundance data for the plot
Community-mean wood density: basal-area-weighted	Community-mean WD, based on each tree’s species WD and weighted by the basal area of each species (e.g., Lewis et al. 2013)	Additionally requires accurate, above-buttress diameter measurement of every individual tree
***Identity-poor metrics***		
Plot-mean wood density	The mean WD of all trees in the plot, based on species WD with species’ contributions weighted by their abundance or basal area	
Forest-type-mean wood density	The mean value of ‘plot-mean wood density’ averaged across contributing plots in the forest type	In the case of the Tambopata landscape, computed separately for Altura and Bajio forest types^a^
Landscape (Tambopata-wide) mean wood density	The mean value of ‘forest-type-mean wood density’, averaged across contributing forest types in the landscape	In the case of the Tambopata landscape, the mean of the mean values for Altura and Bajio forests^a^
Amazon-mean wood density	The mean value of ‘plot-mean wood density’, averaged across contributing plots in Amazonia	Published wood density values from plots across Amazonia (Mitchard et al. 2014)

^a^*Altura* and *Bajio* forest types represent the two major units within the Tambopata landscape. The folk nomenclature used here corresponds to geomorphical units (erosional, depositional) and chronological units (Pleistocene, Holocene). See text for details

Then, to explore the links between composition, structure, and function at Amazon scale we use the latest published data from the RAINFOR long-term plot network (Malhi et al. 2002; Peacock et al. 2007). This includes plots monitored for as long as 30 years (Johnson et al. 2016), with standardised protocols applied to field data collection (Phillips et al. 2010) and data management (Lopez-Gonzalez et al. 2011). This enables us to address for the first time the relationships between forest functional *composition* (WD), forest *structure* (AGB), and forest *dynamics* (stem mortality, AGB mortality) in one analysis. We do this at pan-Amazon level, and also for each of the sub-regions of Amazonia defined by geography and substrate origin (Fittkau 1971; Feldpausch et al. 2012): Western Amazonia (Colombia, Ecuador, and Peru), where soils mostly derive from recent Andean deposits; the Brazilian Shield (Bolivia and Brazil); the Guiana Shield (Guyana, French Guiana, Venezuela); and eastern central Amazonia (Brazil), largely comprised of old sedimentary substrates derived from the other three regions (Quesada et al. 2012; Schargel 2011). We thus assess AGB as a function of WD across more than 150 permanent plots distributed across Amazonia and compare these to long-term measured rates of stem turnover and carbon turnover for the same forests. We ask, (4) Does wood density of Amazon forests correlate with AGB at regional and Amazon-wide scales? (5) Is mean wood density predictable from the long-term dynamics of the same forests?

### Data Analysis

#### Landscape-Level Analyses

To examine whether species wood densities vary with tree size, we calculated the correlation between the diameter of individual trees and their species wood density. This was performed separately for Pleistocene and Holocene forests, pooling data from plots in each landscape. We used nonparametric Kendall’s tau as tree diameter was not normally distributed. We tested whether plot-level mean WD, total basal area and AGB differed between forest types using *t* tests, or Mann–Whitney tests when the response variable was not normally distributed (basal area, AGB). To assess whether landscape-level differences in wood density alter the relationship between basal area and biomass, we used linear models to relate AGB (log-transformed to meet linear model assumptions of normality and homogeneity of variances) to BA, forest type and their interaction, the latter indicating whether the relationship differed between forest types. To quantify the impact of using identity-free estimators of wood density instead of species wood density, we recalculated the AGB of each plot substituting species WD with the different levels of identity-poor WD metrics (Table 1).

#### Amazon-Wide Analyses

We assessed the relationship between biomass and wood density and basal area using bivariate linear regression, fitted both to the pan-Amazon data set and separately to each biogeographic region. We used variation partitioning to identify the independent contributions of each variable to explaining variation in AGB (Legendre and Legendre 2012); linear models were constructed with WD (M1), BA (M2) or both WD density and BA (M3) as explanatory variables, and *R*^*2*^ values extracted. Shared variation due to both variables is calculated as M1_R2_ + M2_R2_ − M3_R2_, which is subtracted from M1_R2_ and M2_R2_ to get the independent effect of each variable. Finally, we used linear regression to assess the bivariate relationships between WD and attributes of forest dynamics, and between AGB and measures of mortality.

## Results

(1) *Wood density varies fivefold among species in Tambopata, with a similar range in both forest types*. Species wood density is only weakly associated with tree size, with correlations between species wood density and diameter slightly stronger in Holocene (Bajio) forests (Kendall’s tau correlation, *τ* = − 0.095, *P* < 0.001) than Pleistocene (Altura) forests (*τ* = − 0.036, *P* < 0.001).

**Fig. 3:**
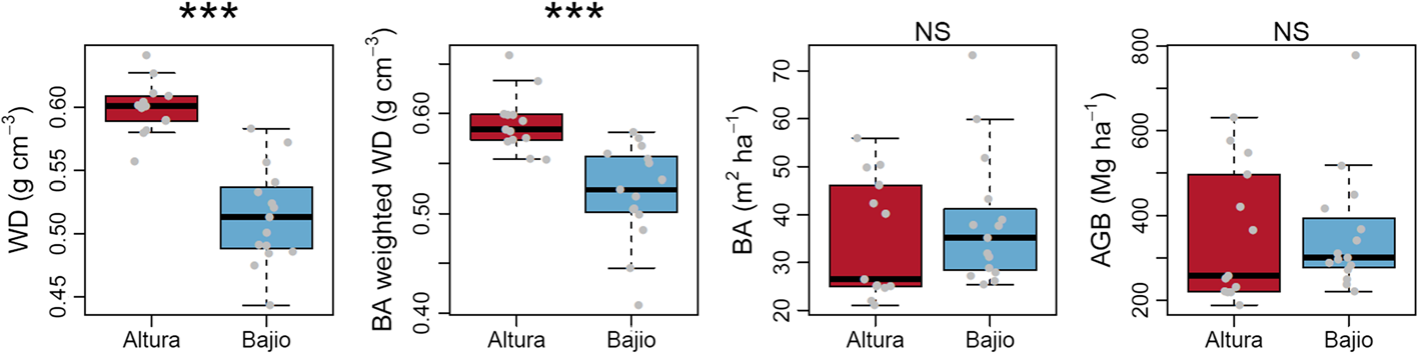
Landscape variation in wood density, basal area and aboveground biomass. Boxplots show variation in each variable within Altura and Bajio forests, with grey points showing values from individual plots (jitter on *x*-axis for presentation purposes only). Differences between Altura and Bajio forests were tested using *t* tests (abundance-weighted wood density (WD), basal-area-weighted wood density) or Mann–Whitney tests (basal area, aboveground biomass), ***P < 0.001; **P < 0.01; *P < 0.05, NS *P* ≥ 0.05

At the plot level (Table S1), there is a marked variation in wood density within landscapes. Abundance-weighted mean wood density was on average 16.6% higher in Altura forests than in Bajio forests (*t* = 7.37, *df* = 22.5, *P* < 0.001, Fig. 3), and basal-area-weighted mean wood density was 13.4% higher (*t* = 4.66, *df* = 23.4, *P* < 0.001, Fig. 3). In contrast, basal area was on average 9.0% higher in Bajio forest plots, but this difference was not statistically significant (Mann–Whitney test, *P* = 0.274, Fig. 3). Aboveground biomass was similar in both forest types (Mann–Whitney test, *P* = 0.387, Fig. 3).

**Fig. 4:**
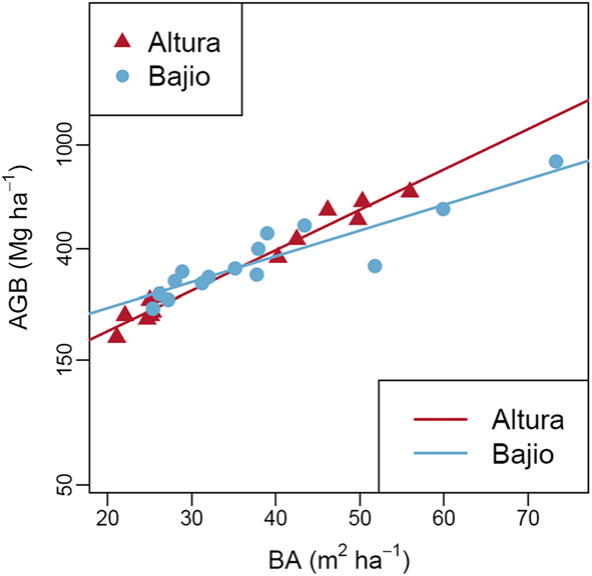
Relationship between stand basal area and aboveground biomass in Altura and Bajio forests. Note that aboveground biomass has been log-transformed to homogenise variances

(2) *Landscape*-*associated differences in wood density greatly alter the relationship between basal area and biomass.* In Altura forests, aboveground biomass increased by 3.6% per 1-m^2^ increase in basal area (linear regression, ln (AGB) = 0.036 ± 0.003 BA, *t* = 12.8, *P* < 0.001, Fig. 4). Yet in Bajio forests, because average wood density was lower, AGB increased by just 2.3% per 1-m^2^ increase in basal area (interaction between forest type and basal area, *β* = − 0.013 ± 0.004, *t* = 3.5, *P* = 0.002, Fig. 4). When analysis is repeated without any one of the three highest basal area outliers, the interaction term remains statistically significant.

Results were unaffected by plot size. Differences in wood density between Altura and Bajio forests remained when restricting analysis to plots of ≥ 1 ha (*t* = 5.15, *df* = 7.0, *P* = 0.001), and the difference in the basal area–biomass relationship between forest types remained statistically significant when allowing it to be scale-dependent (no interaction between plot size and basal area, *t* = 0.05, *P* = 0.638; strong interaction between basal area and forest type, *t* = 3.49, *P* = 0.002).

(3) *Ignoring the actual species identity of each tree biases estimates of forest biomass* (Table S2). Using plot-mean wood density for all trees instead of their species-specific wood density (i.e. representing a situation where we have perfect knowledge of forest-wide spatial variation in wood density but no knowledge of individual identities) results in a mean error in estimating aboveground biomass of 15.0 ± 2.5 Mg ha^−1^ (~ 4.3% of AGB), with a maximum error of 39.8 Mg ha^−1^ (~ 11.9% of AGB) (Table S2). The maximum error was 77.1 Mg ha^−1^ (~ 17% of AGB). This bias itself varies between forest types, being negative for the Bajio forests but not for the Altura forests (Table S2).

**Fig. 5:**
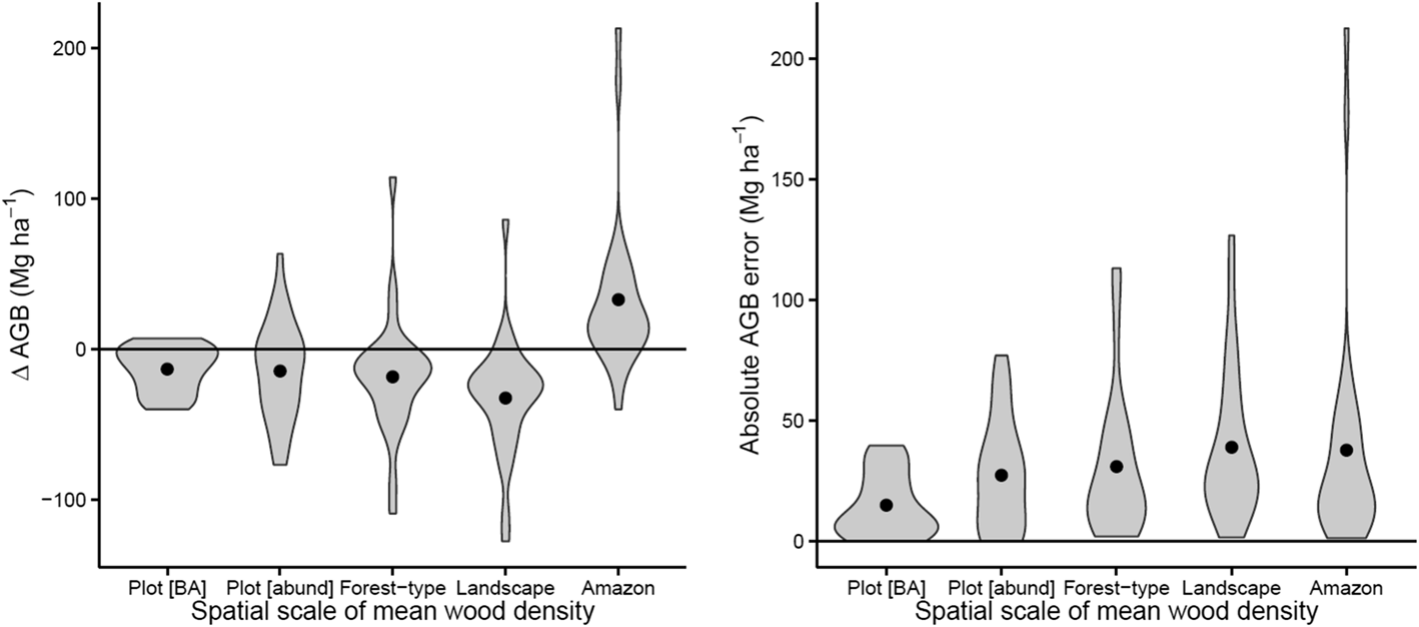
Error in stand-level aboveground biomass estimates when using wood density means calculated as plot, forest type, landscape, and Amazon-wide scales, rather than the actual species values. Violin plots illustrate the distribution of values among plots, while points show the mean error across plots. Note the differences also between abundance-weighted WD and basal-area-weighted WD: the latter clearly entails less bias

Compared to estimates based on species’ wood densities, when values were substituted with average wood density, all these ‘identity-free’ estimates of AGB had error and bias. Both the absolute error and bias increased with the spatial scale of the averaging process (Fig. 5). In particular, absolute bias increased markedly when moving from using a plot- or forest-type-mean wood density to a landscape or Amazon-wide mean wood density (e.g., absolute error was 26.6% higher when landscape-mean wood density was applied instead of the forest-type-mean). Yet even plot-level mean values introduce uncertainty and bias to the forest biomass estimates.

**Fig. 6:**
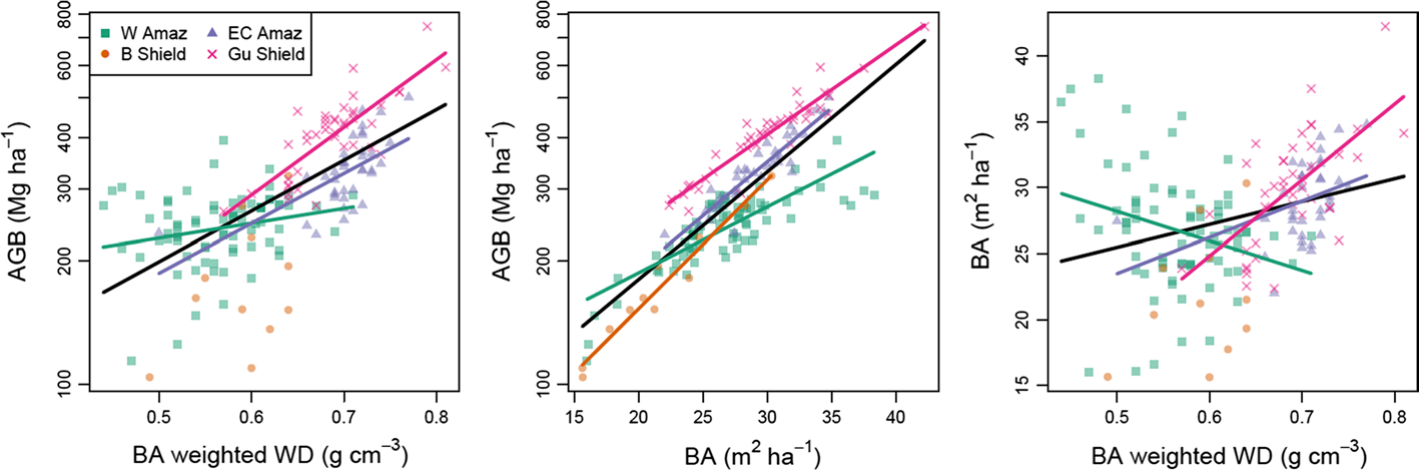
Amazon and regional relationships between basal-area-weighted wood density, biomass, and basal area. For each variable pair, regression models were fitted across the whole data set and for each region. Regions are Western Amazon, Brazilian Shield, East-Central Amazon, Guyana Shield, following Feldpausch et al. 2012. Statistically significant relationships are plotted. Note that regression models with basal-area-weighted wood density predict Amazon biomass with much greater fidelity than simple relationships with basal area alone (Tables S3, S4). Model coefficients are given in Table S5

**Fig. 7:**
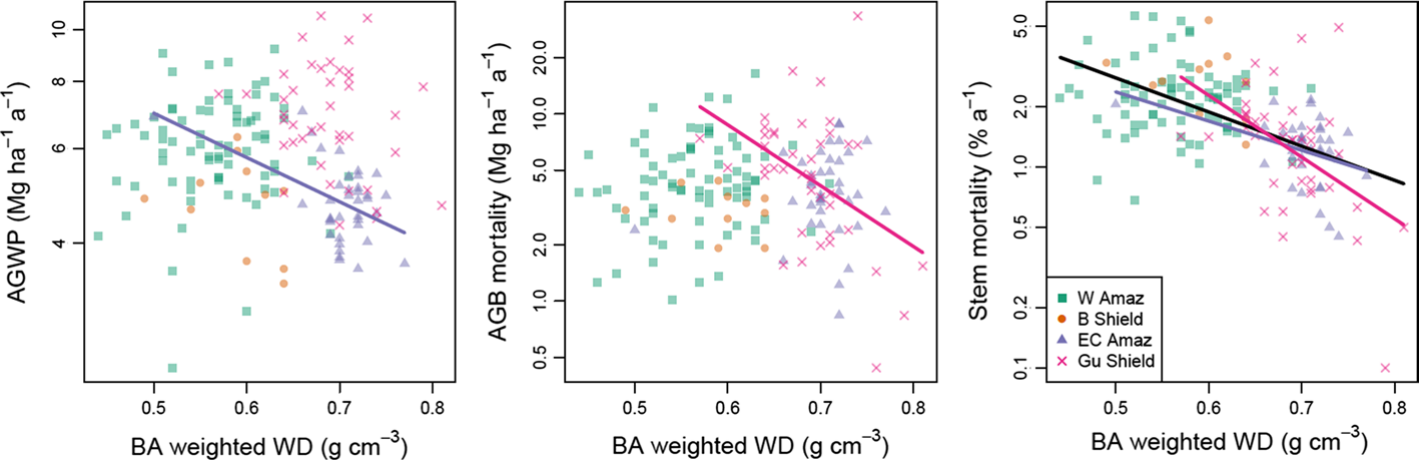
Amazon and regional relationship between forest dynamic processes and wood density. Regression models were fitted across the whole data set and for each region. Statistically significant relationships are plotted. Model coefficients are given in Table S5

**Fig. 8:**
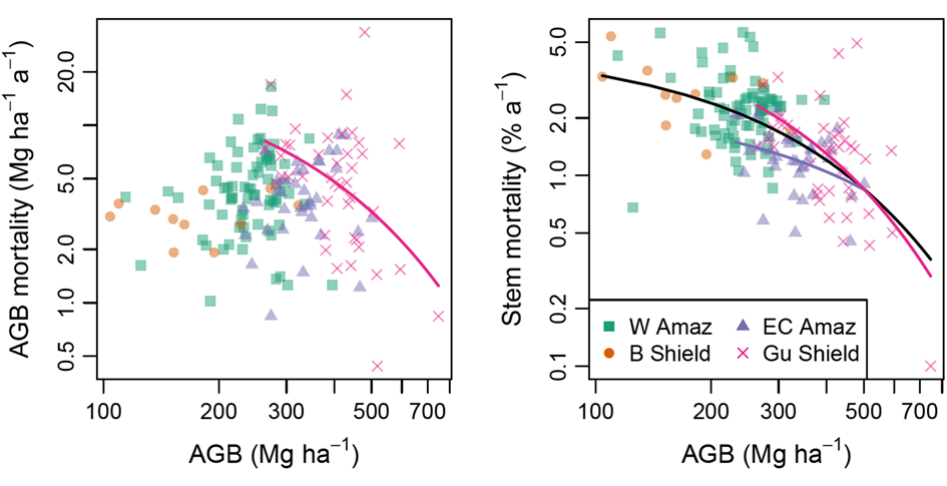
Amazon and regional relationship between forest mortality and AGB. Regression models were fitted for the whole data set and for each region. Statistically significant relationships are plotted. Note the close similarity with the centre and right panels of Fig. 7: species wood density strongly determines biomass and is closely associated with the rate at which individual trees die (figures adapted from Fig. 8 in Johnson et al. 2016). Model coefficients are given in Table S5

(4) *Across the Amazon basin, low forest biomass is strongly associated with both low wood density and low basal area* (Fig. 6). This is of course unsurprising, given that wood density and stem size are used to calculate tree biomass, but these Amazon-basin associations are worth noting especially given varying patterns at sub-regional scales.

(5) *The relationships between basal area, wood density and forest AGB vary, with different slopes and intercepts among regions*. In particular for western Amazonia, relationships are different to the other regions. There are also correlations between stand wood density and basal area, but these are weak and variable among regions (Fig. 6).

(6) Neither the rate of biomass production nor that of its loss is clearly associated with basal-area-weighted wood density. Thus, the *species traits of Amazon forests do not strongly control the rate at which carbon is being cycled by the forest* (Fig. 7, and see Fauset et al. 2019). Yet they are associated with the rates are which *individual trees* are cycled—stem mortality rates are clearly linked to the wood density of the forest, confirming that the lower the stand-level wood density is, the more rapidly the trees die (Fig. 7).

(7) *These relationships between wood density and forest dynamics propagate through to clear relationships between biomass and forest dynamics*. Thus, while standing biomass is not obviously associated with the rate at which wood biomass dies (Fig. 8 left), it is clearly related to the rate at which individual trees die (Fig. 8 right). Lower biomass forests typically have much lower wood density (Fig. 6) and much faster stem turnover (Fig. 8).

## Discussion

Our analysis shows that identity matters. Mapping tropical forest biomass and carbon always benefits from locally validated ground-based measurement of biological variation. In our well-studied Amazon landscape, forest wood density varies within and among landscape geomorphological units. Because relationships between wood density and forest size metrics such as basal area themselves vary, knowledge of forest dimensions and species composition is necessary for estimating tropical biomass. At larger Amazon-wide scales, we also find that lower wood density forests are closely associated with lower AGB and faster stem turnover. Yet within some Amazon regions and particularly western Amazonia, this relationship with forest dynamics breaks down. We explore the implications of these findings here.

In the specific case of Tambopata, the impacts of size and identity on mature-forest biomass are decoupled: while wood density is ≈ 15% greater in the erosional Pleistocene landscape, basal area is equal or greater in depositional Holocene landscapes. As a result, while aboveground biomass is similar in both landscapes, *not* accounting for species effects (i.e. simply assuming a uniform wood density) would substantially underestimate AGB in erosional landscapes. Indeed, *all ‘identity*-*free’ approaches that ignore the floristic variation within these forests lead to biased estimates of forest biomass*. Notably, using a landscape-level Tambopata-wide mean wood density results in equally poor estimates of AGB as using an Amazon-wide mean wood density, although the sign of the errors is reversed. In this landscape, very good, *forest*-*type-specific* floristic knowledge is essential to estimate AGB with an error (bias) of less than 10%. While forest-type- and plot-mean wood density values give performance gains, the error (bias) on both is still 15–30 Mg AGB ha^−1^ compared with the baseline ‘identity-rich’ state in which *every tree in each plot is botanically identified and taxon*-*specific wood density values are used for every tree*. This bias itself varies between forest types, being negative for the Bajio forests but variable for the Altura forests, indicating that in the former canopy trees have greater wood density than understorey trees but not in the latter. This underlines the value of accounting for size and identifying all trees—large *and* small—to reduce bias and uncertainty in forest-level biomass estimation. Our results show that landscape-level mapping of tropical forest biomass requires both tree-level and landscape-level knowledge of biodiversity. Future analyses should account for this and also assess the extent to which accounting for within-species tree level and environmental variation in wood density (e.g., Patiño et al. 2009; Baraloto et al. 2011; Fortunel et al. 2014) might further improve the fidelity of AGB retrieval.

At larger scales, identity also matters: there are large differences in stand-level wood density across Amazonia. Basal-area-weighted wood density varies by 80% between the lowest and highest 1-ha plot values, and even within individual sub-regions it varies by as much as 40%. Low wood density Amazon forests have faster stem turnover, confirming that lower stand-level wood density is associated with more rapid rates of tree death. The relationship is especially marked for stem-level mortality, but much less so for biomass mortality. A key feature of stand-level wood density variation therefore is that it reflects forest dynamics and especially the rate of turnover in the tree population. However, the pan-Amazon associations between wood density and biomass, and wood density and mortality break down at some scales. In some regions (e.g., western Amazonia), variation in the size class distribution among forests may be more important than variation in mean wood density for determining variation in AGB (Fig. 6), but in most regions and across Amazonia, mean wood density is a major determinant of biomass.

### Towards Integrating Species Effects into AGB Mapping

Currently, few attempts to map tropical forest biomass and carbon fully account for species effects—either because they are assumed to be unimportant, or else because ground-level data needed to parameterise and validate them is lacking. Yet foresters have long known that species impact on tropical biomass. An extensive compilation from the last century (Fearnside 1997) suggested that wood density in the Brazilian Amazon varies by 25% across forest types (from 0.60 to 0.75 g cm^−3^), but as this is based on forestry surveys with imprecise identification it contains considerable uncertainty. A recent pan-tropical assessment of forest structure confirmed great variation of wood density within each continent (Sullivan et al. 2017, Fig. S16). This suggests that biodiversity-driven variation in wood density is a pervasive and multi-scalar feature of all tropical forests. Since sufficient inventory-based evidence based on botanical identification now exists to show that species composition matters biome-wide for biomass, we here compiled mean values where we could source *well*-*identified, well*-*measured plot data where basal*-*area weighting has been consistently applied* (Table 2).

**Table 2:** Multi-scale, basal-area-weighted mean community wood density for old-growth forests across the lowland tropical forest biome. Data assembled from the peer-reviewed ecological literature, from scales of 10^0^ to 10^10^ hectares. *All values are basal*-*area-weighted and computed for each plot accounting for taxon*-*specific wood density*^a^. Thus, basal-area-weighted community-level WD of each plot was estimated as Σ BA_*ij*_ × WD_*i*_, where BA_*ij*_ is the relative basal area of species *i* in plot *j*, and WD_*i*_ is the mean wood density of species *i*. Values reported here represent the means of wood density from all available forest plots at the appropriate scale. The nested table structure illustrates how even these mean values vary at all scales, including among continents, among regions and nations, among landscapes within nations, and among forest types within landscapes within nations. Note how the scale at which WD is computed always matters. The best mean WD value to apply will depend on the spatial resolution of the remote sensing and mapping

Continent	Tropical forest climate	Region/nation	Landscape/forest type	Value	Source
Pan-tropical mean				0.619	Mean of Africa, Asia, S. America network mean values assembled here^b^
Africa	Moist			0.633 (CI =+ 0.0080, *n* = 260 plots)	Lewis et al. (2013)
		West Africa		0.61	Lewis et al. (2013)
		Central Africa		0.64	*ibid.*
			Monodominant	0.696	*ibid.*
			Mixed	0.627	*ibid.*
		East Africa		0.61	*ibid.*
		West and Central Africa			
			Acrisols	0.609	Lewis et al. (2013)
			Cambisols	0.617	*ibid.*
			White Sand	0.660	*ibid.*
			Swamp	0.728	*ibid.*
		Central African Republic	Mbaiki: deep resource-rich soils	0.51^c^	Gourlet-Fleury et al. (2011)
			Mbaiki: deep resource-poor soils	0.59^c^	*ibid.*
			Mbaiki: physically constrained soils	0.525^c^	*ibid.*
Asia	Moist			0.594 (SD = 0.039, *n* = 71 plots)	Qie et al. (2017)
		Borneo		0.594	*ibid.*
			Old-growth, no edge effects	0.600 (SD = 0.038, *n* = 49 plots	*ibid.*
			Old-growth, edge effects	0.581 (SD = 0.039, *n* = 22 plots)	*ibid.*
		Borneo: Sabah	Sepilok: Alluvial	0.55	Jucker et al. (2018a, b)
			Sepilok: White Sand	0.64	*ibid.*
Central America				0.540 (SD = 0.063, *n* = 5 sites)	This paper, from literature sources
	Wet	Costa Rica	La Selva	0.47^d^	Muller-Landau (2004)
		Panama	Sherman	0.595	Stegen et al. (2009)
	Moist	Panama	Barro Colorado Island	0.51^d^	Muller-Landau (2004)
		Panama	Barro Colorado Island	0.545	Stegen et al. (2009)
	Dry	Panama	Cocoli	0.494	*ibid.*
		Costa Rica	San Emilio	0.614	*ibid.*
South America: Amazonia	Moist	All Amazon		0.629 (SD = 0.081, *n* = 165 plots)	This paper, from RAINFOR data
		Central Amazon		0.703 (SD = 0.041, *n* = 37 plots)	This paper; updating Baker et al. (2004), Mitchard et al. (2014)
		Brazilian Shield		0.591 (SD = 0.048, *n* = 11 plots)	*ibid.*
		Guyana Shield		0.688 (SD = 0.048, *n* = 41 plots)	*ibid.*
			Paracou: Terra Firme and Alluvial	0.67^e^	Baraloto et al. (2011)
			Paracou: White Sand	0.72	*ibid.*
		Western Amazon		0.566 (SD = 0.056, *n* = 76 plots)	This paper, updating Baker et al. (2004), Mitchard et al. (2014)
		Ecuador	Yasuni: Terra Firme	0.588	Stegen et al. (2009)
		Peru	Loreto: Terra Firme and Flooded	0.62^e^	Baraloto et al. (2011)
			Loreto: White Sand	0.64	*ibid.*
		Peru	Tambopata	0.554 (SD = 0.053, *n* = 28 plots)	This paper
			Tambopata: Holocene	0.521 (SD = 0.049, *n* = 15 plots)	*ibid.*
			Tambopata: Pleistocene	0.591 (SD = 0.029, *n* = 13 plots)	*ibid.*
			Tambopata: swamp	0.467 (SD = 0.034, *n* = 2 plots)	*ibid.*

^a^Multi-plot studies and compilations that present community-weighted wood density for tropical forests were only included if values were clearly basal-area-weighted and properly identified. Thus, (1) studies that apparently represent the average wood density of all species or stems in plots or other samples (e.g., ter Steege et al. 2006; Slik et al. 2010, Fortunel et al. 2014) were not included, because weighting by relative contribution to basal area is more likely to approximate the contribution of each species to carbon storage than weighting by its relative frequency or abundance (cf. the large differences in Amazon-dominant species as reported by Fauset et al. 2015 and ter Steege et al. 2013 when evaluated by basal area and when evaluated by stem abundance). Similarly, (2) studies based largely or entirely on vernacular name identifications are excluded, as in diverse tropical forests these are less reliable and precise than botanical identifications (cf. Fearnside 1997 for data and discussion of this). Sullivan et al. 2017 is not listed as a source in this table as data plotted in their Fig S16 are mostly available as continent-level mean values in other recent analyses (Lewis et al. 2013 for Africa, Qie et al. 2017 for Borneo, and the current paper for Amazonia)

^b^The simple unweighted mean of Amazon, Asian, and African moist forest values here from the plot networks across tropical forest Africa (AfriTRON), Asia (T-FORCES), and South America (RAINFOR), where trees ≥ 10 cm d.b.h. and a standard wood density data source (Zanne et al. 2009) were used. No pan-tropical value could be located in previous literature that was clearly based on plot measurements in which trees were identified to species and trees were all measured

^c^Trees ≥ 20 cm d.b.h.; from data plotted in Fig. 2 of Gourlet-Fleury et al. (2011)

^d^Trees > 30 cm d.b.h

^e^From data plotted in Fig. 5 of Baraloto et al. (2011)

Accounting for variation in such forest-wide means can improve biomass estimates. We find differences in intact forest basal-area-weighted wood density of as much as 20% in African, Bornean and Amazon landscapes of 10^1^–10^3^ km^2^, of 20% within 10^5^–10^6^-km^2^ geographic regions in north-west Amazonia and French Guiana, and 10–30% at the continental scale (between 10^7^ km^2^ units) between south-west and north-east Amazonia. The data compiled in Table 2 show that wood density impacts hugely on biomass even at continental levels. For example, applying a pan-tropical wood density mean to Central American forests could result in over-estimating aboveground carbon stocks there by 15%.

Our pan-Amazon results suggest a possible avenue for using technology like LiDAR to indirectly derive the key composition-based property of wood density. Thus, by quantifying tree mortality rates it may be possible to estimate wood density and so improve LiDAR’s ability to estimate AGB. LiDAR is being increasingly used to sense tree biomass mortality (e.g., Espírito-Santo et al. 2014; Leitold et al. 2018). If these estimates can be produced over large-enough spatial and temporal scales to yield time-averaged tree mortality rates, it may be possible to derive proxies for wood density and validate them with plot species-level identifications. This would be a promising angle to explore, for example, with repeat-survey LiDAR data as they become available. Similarly, hyperspectral properties of forest canopies may correlate with wood density and underlying soil conditions, so these hold promise for deriving canopy wood density estimates that can be validated with full forest species-level identifications.

The multi-scale variability in forest wood density means that the next generation of tropical forest carbon maps and models needs to account better for species and functional variation. Mapping at all scales benefits from locally validated, ground-based identification of measured trees. Because most tropical forests are very diverse, this requires highly skilled professional botanists to collect and identify the trees, working in georeferenced plots, measured carefully and more-or-less synchronously with remote sensing measurements. There are currently just a handful of tropical forest landscapes where remote- and ground-based measurements exist with the requisite level of species identification (Chave et al. 2019). We need many more, distributed across key environmental and biodiversity gradients, if tropical forest nations are to realise the potential of remote sensing to help measure and validate their carbon stocks, fluxes, and nationally determined contributions to the Paris Climate Accord.

## Electronic supplementary material

Below is the link to the electronic supplementary material.

Supplementary material 1 (DOCX 81 kb)

## References

[CR1] Asner GP, Martin RE, Knapp DE, Tupayachi R, Anderson CB, Sinca F, Vaughn NR, Llactayo W (2017). Airborne laser-guided imaging spectroscopy to map forest trait diversity and guide conservation. Science.

[CR2] Avitabile V, Herold M, Heuvelink GB, Lewis SL, Phillips OL, Asner GP, Armston J, Ashton PS, Banin L, Bayol N (2016). An integrated pan-tropical biomass map using multiple reference datasets. Glob Change Biol.

[CR3] Baker TR (2018) biodiversity increases the resilience of tropical forests to climate change: implications for conservation policy. In: Rodríguez L, Anderson I (eds) Secretariat of the convention on biological diversity the Lima declaration on biodiversity and climate change: contributions from science to policy for sustainable development. Technical series no. 89, pp 24–31

[CR4] Baker TR, Phillips OL, Malhi Y, Almeida S, Arroyo L, Di Fiore A, Erwin T, Killeen TJ, Laurance SG, Laurance WF (2004). Variation in wood density determines spatial patterns in Amazonian forest biomass. Glob Change Biol.

[CR5] Baker TR, Phillips OL, Laurance WF, Pitman NC, Almeida S, Arroyo L, DiFiore A, Erwin T (2009). Do species traits determine patterns of wood production in Amazonian forests?. Biogeosciences.

[CR6] Baker TR, Pennington RT, Dexter KG, Fine PV, Fortune-Hopkins H, Honorio EN (2017). Maximising synergy among tropical plant systematists, ecologists, and evolutionary biologists. Trends Ecol Evol.

[CR7] Baraloto C, Rabaud S, Molto Q, Blanc L, Fortunel C, Herault B, Davila N, Mesones I, Rios M, Valderrama E, Fine PV (2011). Disentangling stand and environmental correlates of aboveground biomass in Amazonian forests. Glob Change Biol.

[CR8] Brown S, Lugo AE (1990). Tropical secondary forests. J Trop Ecol.

[CR9] Bunker DE, DeClerck F, Bradford JC, Colwell RK, Perfecto I, Phillips OL, Sankaran M, Naeem S (2005). Species loss and aboveground carbon storage in a tropical forest. Science.

[CR10] Chao KJ, Phillips OL, Baker TR, Peacock J, Lopez-Gonzalez G, Vásquez Martínez R, Monteagudo A, Torres-Lezama A (2009). After trees die: quantities and determinants of necromass across Amazonia. Biogeosciences.

[CR11] Chave J, Davies SJ, Phillips OL, Sist P, Schepaschenko D, Armston J, Baker TR, Coomes D (2019). Ground data are essential for biomass remote sensing missions. Surv Geophys.

[CR12] Chave J, Muller-Landau HC, Baker TR, Easdale TA, ter Steege H, Webb CO (2006). Regional and phylogenetic variation of wood density across 2456 neotropical tree species. Ecol Appl.

[CR13] Chave J, Réjou-Méchain M, Búrquez A, Chidumayo E, Colgan MS, Delitti WBC, Duque A (2014). Improved allometric models to estimate the aboveground biomass of tropical trees. Glob Change Biol.

[CR14] Clinebell R, Phillips OL, Gentry AH, Stark N, Zuuring H (1995). Prediction of neotropical woody plant diversity from soil and climatic data. Biodivers Conserv.

[CR15] Coelho de Souza F, Dexter KG, Phillips OL, Brienen RJW, Chave J, Galbraith DR, Lopez Gonzalez G, Monteagudo Mendoza A (2016). Evolutionary heritage influences Amazon tree ecology. Proc R Soc B Biol Sci.

[CR16] Condit R, Pitman N, Leigh EG, Chave J, Terborgh J, Foster RB, Núnez P, Aguilar S, Valencia R (2002). Beta-diversity in tropical forest trees. Science.

[CR17] Conservation International and Foster RB (1994). The Tambopata-Candamo reserved zone of southeastern Perú: a biological assessment.

[CR18] Coomes DA, Dalponte M, Jucker T, Asner GP, Banin LF, Burslem DF, Lewis SL, Nilus R, Phillips OL (2017). Area-based vs tree-centric approaches to mapping forest carbon in Southeast Asian forests from airborne laser scanning data. Remote Sens Environ.

[CR19] Cosme LH, Schietti J, Costa FR, Oliveira RS (2017). The importance of hydraulic architecture to the distribution patterns of trees in a central Amazonian forest. New Phytol.

[CR20] Draper F, Baraloto C, Brodrick P, Phillips OLB, Vásquez R, Honorio Coronado E, Baker T et al. (2019) Imaging spectroscopy predicts variable distance decay across contrasting Amazonian tree communities. J Ecol (in press)

[CR21] Duncanson L, Armston J, Disney M, Avitabile V, Barbier N, Calders K, Carter S, Chave J et al The importance of global land product validation: towards a standardized protocol for aboveground biomass. Surv Geophys (in press) 10.1007/s10712-019-09538-8 PMC6647371 31395994

[CR22] Espírito-Santo FD, Gloor M, Keller M, Malhi Y, Saatchi S, Nelson B, Junior RC, Pereira C, Lloyd J (2014). Size and frequency of natural forest disturbances and the Amazon forest carbon balance. Nat Commun.

[CR23] Fauset S, Johnson MO, Gloor M, Baker TR, Monteagudo A, Brienen RJ, Feldpausch TR (2015). Hyperdominance in Amazonian forest carbon cycling. Nat Commun.

[CR24] Fauset SF, Gloor M, Fyllas N, Phillips OL, Asner GP, Baker T, Bentley L, Brienen R (2019). Individual-based modelling of Amazon forests suggests that climate controls productivity while traits control demography. Front Earth Sci.

[CR25] Fearnside PM (1997). Wood density for estimating forest biomass in Brazilian Amazonia. For Ecol Manag.

[CR26] Feldpausch TR, Lloyd J, Lewis SL, Brienen RJ, Gloor M, Monteagudo Mendoza A, Lopez-Gonzalez G, Banin L (2012). Tree height integrated into pantropical forest biomass estimates. Biogeosciences.

[CR27] Fittkau EJ (1971). Esboco de uma divisão ecologica da regiåo amazónica.. Proc Symp Biol Trop Amaz Florencia y Leticia.

[CR28] Fortunel C, Ruelle J, Beauchêne J, Fine PV, Baraloto C (2014). Wood specific gravity and anatomy of branches and roots in 113 Amazonian rainforest tree species across environmental gradients. New Phytol.

[CR29] Fyllas NM, Patiño S, Baker TR, Bielefeld Nardoto G, Martinelli LA, Quesada CA, Paiva R, Schwarz M (2009). Basin-wide variations in foliar properties of Amazonian forest: phylogeny, soils and climate. Biogeosciences.

[CR30] Gentry AH (1988). Changes in plant community diversity and floristic composition on environmental and geographical gradients. Ann Mo Bot Gard.

[CR31] Good P, Bamber J, Halladay K, Harper AB, Jackson LC, Kay G, Kruijt B, Lowe JA (2018). Recent progress in understanding climate thresholds. Prog Phys Geogr Earth Environ.

[CR32] Goodman RC, Phillips OL, Baker TR (2012). Tropical forests: tightening up on tree carbon estimates. Nature.

[CR33] Goodman RC, Phillips OL, Baker TR (2014). The importance of crown dimensions to improve tropical tree biomass estimates. Ecol Appl.

[CR34] Goodman RC, Phillips OL, del Castillo Torres D, Freitas L, Cortese ST, Monteagudo A, Baker TR (2014). Amazon palm biomass and allometry. For Ecol Manag.

[CR35] Gourlet-Fleury S, Rossi V, Rejou-Mechain M, Freycon V, Fayolle A, Saint-André L, Cornu G, Gérard J, Sarrailh JM, Flores O, Baya F (2011). Environmental filtering of dense-wooded species controls above-ground biomass stored in African moist forests. J Ecol.

[CR36] Hietz P, Rosner S, Hietz-Seifert U, Wright SJ (2017). Wood traits related to size and life history of trees in a Panamanian rainforest. New Phytol.

[CR37] Higgins MA, Ruokolainen K, Tuomisto H, Llerena N, Cardenas G, Phillips OL, Vásquez R, Räsänen M (2011). Geological control of floristic composition in Amazonian forests. J Biogeogr.

[CR38] Honorio Coronado EN, Baker TR, Phillips OL, Pitman NC, Pennington RT, Vasquez Martinez R, Monteagudo A (2009). Multi-scale comparisons of tree composition in Amazonian terra firme forests. Biogeosciences.

[CR39] Johnson MO, Galbraith D, Gloor M, De Deurwaerder H, Guimberteau M, Rammig A, Thonicke K (2016). Variation in stem mortality rates determines patterns of above-ground biomass in Amazonian forests: implications for dynamic global vegetation models. Glob Change Biol.

[CR40] Jucker T, Asner GP, Dalponte M, Brodrick PG, Philipson CD, Vaughn NR, Teh YA, Brelsford C, Burslem DF, Deere NJ, Ewers RM (2018). Estimating aboveground carbon density and its uncertainty in Borneo’s structurally complex tropical forests using airborne laser scanning. Biogeosciences.

[CR41] Jucker T, Bongalov B, Burslem DF, Nilus R, Dalponte M, Lewis SL, Phillips OL, Qie L, Coomes DA (2018). Topography shapes the structure, composition and function of tropical forest landscapes. Ecol Lett.

[CR42] Kalliola R, Salo J, Puhakka M, Rajasilta M, Häme T, Neller RJ, Räsänen ME, Arias WD (1992). Upper Amazon channel migration. Naturwissenschaften.

[CR43] Lawrence A, Phillips OL, Ismodes AR (2005). Local values for harvested forest plants in Madre de Dios, Peru: towards a more contextualised interpretation of quantitative ethnobotanical data. Biodivers Conserv.

[CR44] Legendre P, Legendre LF (2012). Numerical ecology.

[CR45] Leitold V, Morton DC, Longo M, dos-Santos MN, Keller M, Scaranello M (2018). El Niño drought increased canopy turnover in Amazon forests. New Phytol.

[CR46] Levis C, Costa FR, Bongers F, Peña-Claros M, Clement CR, Junqueira AB, Neves EG (2017). Persistent effects of pre-Columbian plant domestication on Amazonian forest composition. Science.

[CR47] Lewis SL, Sonké B, Sunderland T, Begne SK, Lopez-Gonzalez G, van der Heijden GMF, Phillips OL (2013). Above-ground biomass and structure of 260 African tropical forests. Philos Trans R Soc B Biol Sci.

[CR48] Lopez-Gonzalez G, Lewis SL, Burkitt M, Phillips OL (2011). ForestPlots.net: a web application and research tool to manage and analyse tropical forest plot data. J Veg Sci.

[CR49] Lopez-Gonzalez G, Sullivan MJP, Baker TR (2015) BiomasaFP: tools for analysing data downloaded from ForestPlots.net. R package version 1.1

[CR50] Malhi Y, Phillips OL, Lloyd J, Baker T, Wright J, Almeida S, Arroyo L, Frederiksen T, Grace J, Higuchi N (2002). An international network to monitor the structure, composition and dynamics of Amazonian forests (RAINFOR). J Veg Sci.

[CR51] Malhi Y, Wood D, Baker TR, Wright J, Phillips OL, Cochrane T, Meir P, Chave J, Almeida S, Arroyo L (2006). The regional variation of aboveground live biomass in old-growth Amazonian forests. Glob Change Biol.

[CR52] Malhi Y, Farfán Amézquita F, Doughty CE, Silva-Espejo JE, Girardin CA, Metcalfe DB, Aragão LE (2014). The productivity, metabolism and carbon cycle of two lowland tropical forest plots in south-western Amazonia, Peru. Plant Ecol Divers.

[CR53] Minh DH, Le Toan T, Rocca F, Tebaldini S, d’Alessandro MM, Villard L (2014). Relating P-band synthetic aperture radar tomography to tropical forest biomass. IEEE Trans Geosci Remote Sens.

[CR54] Mitchard ETA, Feldpausch TR, Brienen RJW, Lopez-Gonzalez G, Monteagudo A, Baker TR (2014). Markedly divergent estimates of Amazon forest carbon density from ground plots and satellites. Glob Ecol Biogeogr.

[CR55] Muller-Landau HC (2004). Interspecific and inter-site variation in wood specific gravity of tropical trees. Biotropica.

[CR56] Osher LJ, Buol SW (1998). Relationship of soil properties to parent material and landscape position in eastern Madre de Dios, Peru. Geoderma.

[CR57] Pallqui NC, Monteagudo A, Phillips OL, Lopez-Gonzalez G, Cruz L, Galiano W, Chavez W, Vasquez R (2014). Dinámica, biomasa aérea y composición florística en parcelas permanentes Reserva Nacional Tambopata, Madre de Dios, Perú. Rev Peru Biol.

[CR58] Palmero P (2004) Characterising the fine-scale pattern of Amazonian forest type using multi-source Earth observation data. Ph.D. thesis, University of Leeds, UK

[CR59] Patiño S, Lloyd J, Paiva R, Baker TR, Quesada CA, Mercado LM (2009). Branch xylem density variations across the Amazon Basin. Biogeosciences.

[CR60] Peacock J, Baker TR, Lewis SL, Lopez-Gonzalez G, Phillips OL (2007). The RAINFOR database: monitoring forest biomass and dynamics. J Veg Sci.

[CR86] Peru Ministerio de Ambiente (2015) Mapa del Patrimonio Forestal Nacional. https://sinia.minam.gob.pe/mapas/mapa-patrimonio-forestal-nacional

[CR61] Phillips OL (2018) Recent changes in Amazon forest biomass and dynamics. In: Rodríguez L, Anderson I (eds) The Lima declaration on biodiversity and climate change: contributions from science to policy for sustainable development. Technical series no. 89. Secretariat of the Convention on Biological Diversity, pp 32–41

[CR62] Phillips O, Miller JS (2002). Global patterns of plant diversity: Alwyn H. Gentry’s forest transect data set. Monogr Syst Bot.

[CR63] Phillips OL, Núñez Vargas P, Monteagudo A, Peña Cruz A, Chuspe Zans ME, Galiano Sánchez W, Yli-Halla M, Rose S (2003). Habitat association among Amazonian tree species: a landscape-scale approach. J Ecol.

[CR64] Phillips OL, Baker TR, Arroyo L, Higuchi N, Killeen TJ, Laurance WF, Lewis SL, Lloyd J, Malhi Y, Monteagudo A (2004). Pattern and process in Amazon tree turnover, 1976–2001. Philos Trans R Soc Lond Ser B Biol Sci.

[CR65] Phillips OL, Rose S, Monteagudo Mendoza A, Núñez Vargas P (2006). Resilience of southwestern Amazon forests to anthropogenic edge effects. Conserv Biol.

[CR66] Phillips OL, Baker TR, Brienen R, Feldpausch TR (2010) Field manual for plot establishment and remeasurement. http://www.geog.leeds.ac.uk/projects/rainfor

[CR67] Phillips OL, Brienen RW (2017). Carbon uptake by mature Amazon forests has mitigated Amazon nations’ carbon emissions. Carbon Balance Manag.

[CR68] Qie L, Lewis SL, Sullivan MJ, Lopez-Gonzalez G, Pickavance GC, Sunderland T, Ashton P, Hubau W (2017). Long-term carbon sink in Borneo’s forests halted by drought and vulnerable to edge effects. Nat Commun.

[CR69] Quesada CA, Phillips OL, Schwarz M, Czimczik CI, Baker TR, Patiño S, Fyllas NM, Hodnett MG, Herrera R (2012). Basin-wide variations in Amazon forest structure and function are mediated by both soils and climate. Biogeosciences.

[CR70] Räsänen M, Neller R, Salo J, Jungner H (1992). Recent and ancient fluvial deposition systems in the Amazonian foreland basin, Peru. Geol Mag.

[CR71] Salo J, Kalliola R, Häkkinen I, Mäkinen Y, Niemelä P, Puhakka M, Coley PD (1986). River dynamics and the diversity of Amazon lowland forest. Nature.

[CR72] Schargel R (2011). Una resena de la geografıa fısica de Venezuela, con enfasis en los suelos. BioLlania Edicion Especial.

[CR73] Slik JW, Aiba SI, Brearley FQ, Cannon CH, Forshed O, Kitayama K, Nagamasu H, Nilus R (2010). Environmental correlates of tree biomass, basal area, wood specific gravity and stem density gradients in Borneo’s tropical forests. Glob Ecol Biogeogr.

[CR74] Stegen JC, Swenson NG, Valencia R, Enquist BJ, Thompson J (2009). Above-ground forest biomass is not consistently related to wood density in tropical forests. Glob Ecol Biogeogr.

[CR75] Sullivan MJ, Talbot J, Lewis SL, Phillips OL, Qie L, Begne SK, Chave J (2017). Diversity and carbon storage across the tropical forest biome. Sci Rep.

[CR76] Sullivan MJP, Lewis SL, Hubau W, Qie L, Baker TR, Banin LF, Chave J (2018). Field methods for sampling tree height for tropical forest biomass estimation. Methods Ecol Evol.

[CR77] ter Steege H, Pitman NC, Phillips OL, Chave J, Sabatier D, Duque A, Molino JF, Prévost MF, Spichiger R, Castellanos H, Von Hildebrand P (2006). Continental-scale patterns of canopy tree composition and function across Amazonia. Nature.

[CR78] ter Steege H, Pitman NC, Sabatier D, Baraloto C, Salomão RP, Guevara JE, Phillips OL, Castilho CV (2013). Hyperdominance in the Amazonian tree flora. Science.

[CR79] Tuomisto H, Ruokolainen K, Kalliola R, Linna A, Danjoy W, Rodriguez Z (1995). Dissecting Amazonian biodiversity. Science.

[CR80] Turner IM (2001). The ecology of trees in the tropical rain forest.

[CR81] Vásquez R, Rojas R, Monteagudo AM, Valenzuela LG, Huamantupa I (2018) Catalogo de los Arboles del Perú. Q’ueña Revista de la Sociedad Botánica del Cusco 9(1), número especial

[CR82] Vicuña Miñano E, Baker TB, Banda K, Honorio Coronado E, Monteagudo A (2018). El sumidero de carbono en los bosques primarios Amazónicos es una oportunidad para lograr la sostenibilidad de su conservación. Folia Amazónica.

[CR83] Watson JEM, Evans T, Venter O, Williams B, Tulloch A, Stewart C (2018). The exceptional value of intact forest ecosystems. Nat Ecol Evol.

[CR84] Zanne AE, Lopez-Gonzalez G, Coomes DA, Ilic J, Jansen S, Lewis SL, Miller RB, Swenson NG, Wiemann MC, Chave J (2009) Global wood density database. Dryad. http://hdl. handle.net/10255/dryad235

[CR85] Zolkos SG, Goetz SJ, Dubayah R (2013). A meta-analysis of terrestrial aboveground biomass estimation using lidar remote sensing. Remote Sens Environ.

