# Supplementary material for: Species Matter: Wood Density Influences Tropical Forest Biomass at Multiple Scales
Source: Surv Geophys. 2019 Jun 3;40(4):913–35. doi: 10.1007/s10712-019-09540-0 (PMC6647473; doi:10.1007/s10712-019-09540-0)
Supplement: Supplementary file 1 — Supplementary material 1 (DOCX 81 kb) [file 10712_2019_9540_MOESM1_ESM.docx]

**APPENDIX - Supporting information**

**Table S1.** Aboveground biomass, stand basal area and wood density in Altura and Bajio forests sampled in the lower Tambopata region. AGB was estimated using the Chave et al. (2014) allometric equation, with forest type height-diameter models used to estimate tree height. The biomass of palms was estimated using the allometric equation developed by Goodman et al. 2014b.

| Plot | Type | Census | AGB (Mg ha^-1^) | BA (m^2^ ha^-1^) | WD (g cm^-3^) | WD _basal area weighted_ (g cm^-3^) |
| --- | --- | --- | --- | --- | --- | --- |
| CJC-05 | Altura | 1 | 222.6 | 25.1 | 0.58 | 0.57 |
| CJC-06 | Altura | 1 | 605.0 | 50.3 | 0.60 | 0.63 |
| CJC-07 | Altura | 1 | 515.9 | 49.8 | 0.60 | 0.57 |
| CJC-08 | Altura | 1 | 395.4 | 40.3 | 0.56 | 0.55 |
| CLT-01 | Altura | 1 | 658.5 | 55.9 | 0.59 | 0.58 |
| CLT-02 | Altura | 1 | 561.1 | 46.2 | 0.63 | 0.66 |
| CLT-04 | Altura | 1 | 183.6 | 21.1 | 0.61 | 0.59 |
| CLT-06 | Altura | 1 | 215.5 | 24.7 | 0.60 | 0.60 |
| CLT-08 | Altura | 1 | 229.2 | 25.4 | 0.64 | 0.58 |
| TAM-05 | Altura | 7 | 254.1 | 25.0 | 0.61 | 0.60 |
| TAM-07 | Altura | 3 | 261.2 | 26.5 | 0.60 | 0.58 |
| TAM-08 | Altura | 1 | 222.2 | 22.1 | 0.58 | 0.60 |
| TAM-23 | Altura | 1 | 433.3 | 42.5 | 0.59 | 0.55 |
|  |  |  |  |  |  |  |
| CJC-09 | Bajio | 1 | 299.9 | 28.0 | 0.58 | 0.58 |
| CJC-10 | Bajio | 1 | 343.1 | 51.8 | 0.44 | 0.41 |
| CLT-03 | Bajio | 1 | 398.3 | 37.9 | 0.51 | 0.55 |
| CLT-05 | Bajio | 1 | 293.7 | 31.2 | 0.54 | 0.52 |
| CLT-07 | Bajio | 1 | 326.7 | 28.9 | 0.56 | 0.56 |
| CLT-09 | Bajio | 1 | 862.0 | 73.3 | 0.49 | 0.51 |
| INF-01 | Bajio | 1 | 311.5 | 32.0 | 0.47 | 0.50 |
| TAM-01 | Bajio | 4 | 236.1 | 25.4 | 0.52 | 0.52 |
| TAM-02 | Bajio | 13 | 255.3 | 27.2 | 0.52 | 0.53 |
| TAM-03 | Bajio | 2 | 316.8 | 37.7 | 0.49 | 0.48 |
| TAM-06 | Bajio | 11 | 334.7 | 35.2 | 0.48 | 0.50 |
| TAM-09 | Bajio | 3 | 268.2 | 26.2 | 0.57 | 0.57 |
| TAM-20 | Bajio | 1 | 457.3 | 39.0 | 0.49 | 0.55 |
| TAM-21 | Bajio | 1 | 567.1 | 59.9 | 0.50 | 0.45 |
| TAM-22 | Bajio | 1 | 487.9 | 43.4 | 0.53 | 0.58 |

**Table S2.** Change in estimates of aboveground biomass when using wood density values averaged across plots, forest types, landscapes, and the whole Amazon, rather than the values derived from the actual species identity of each tree. Basal-area weighted wood density values are used for each level.

| Plot | Type | ΔAGB_WD_plot_ | | ΔAGB_WD_type_ | | ΔAGB_WD_landscape_ | | ΔAGB_WD_amazon_ | |
| --- | --- | --- | --- | --- | --- | --- | --- | --- | --- |
|  |  | Mg ha^-1^ | % | Mg ha^-1^ | % | Mg ha^-1^ | % | Mg ha^-1^ | % |
| CJC-05 | Altura | 1.3 | 0.6 | -9.0 | -4.1 | -17.6 | -7.9 | 21.7 | 9.8 |
| CJC-06 | Altura | -9.7 | -1.6 | -88.1 | -14.6 | -109.5 | -18.1 | -11.1 | -1.8 |
| CJC-07 | Altura | 5.4 | 1.0 | -17.0 | -3.3 | -37.0 | -7.2 | 55.0 | 10.7 |
| CLT-01 | Altura | -7.0 | -1.1 | -47.3 | -7.2 | -73.1 | -11.1 | 46.0 | 7.0 |
| CLT-02 | Altura | -18.9 | -3.4 | -108.9 | -19.4 | -127.9 | -22.8 | -40.5 | -7.2 |
| CLT-04 | Altura | 0.6 | 0.3 | -14.1 | -7.7 | -21.4 | -11.7 | 12.1 | 6.6 |
| CLT-06 | Altura | 0.6 | 0.3 | -18.1 | -8.4 | -26.4 | -12.2 | 11.8 | 5.5 |
| CLT-08 | Altura | 6.8 | 3.0 | -8.6 | -3.8 | -18.0 | -7.9 | 25.3 | 11.1 |
| TAM-05 | Altura | 0.7 | 0.3 | -22.3 | -8.8 | -32.2 | -12.7 | 13.3 | 5.2 |
| TAM-07 | Altura | 7.2 | 2.8 | -9.7 | -3.7 | -20.4 | -7.8 | 29.0 | 11.1 |
| TAM-08 | Altura | -11.8 | -5.3 | -30.5 | -13.7 | -38.3 | -17.2 | -2.3 | -1.0 |
| TAM-23 | Altura | 3.5 | 0.8 | -4.0 | -0.9 | -22.3 | -5.2 | 61.9 | 14.3 |
|  |  |  |  |  |  |  |  |  |  |
| CJC-08 | Bajio | -1.8 | -0.5 | -7.8 | -2.1 | -23.0 | -6.2 | 47.2 | 12.8 |
| CJC-09 | Bajio | -35.6 | -11.9 | -48.6 | -16.2 | -57.1 | -19.0 | -17.9 | -6.0 |
| CJC-10 | Bajio | -9.1 | -2.7 | 1.3 | 0.4 | -0.6 | -0.2 | 7.8 | 2.3 |
| CLT-03 | Bajio | -39.2 | -9.8 | -45.2 | -11.3 | -59.1 | -14.8 | 4.9 | 1.2 |
| CLT-05 | Bajio | -8.7 | -2.9 | 1.8 | 0.6 | -10.4 | -3.5 | 45.5 | 15.5 |
| CLT-07 | Bajio | -27.4 | -8.4 | -34.5 | -10.6 | -45.8 | -14.0 | 6.4 | 2.0 |
| CLT-09 | Bajio | -21.1 | -2.4 | 41.0 | 4.8 | 3.2 | 0.4 | 177.2 | 20.6 |
| INF-01 | Bajio | -34.3 | -11.0 | -12.9 | -4.1 | -24.3 | -7.8 | 28.1 | 9.0 |
| TAM-01 | Bajio | -22.4 | -9.5 | -13.2 | -5.6 | -21.6 | -9.2 | 17.2 | 7.3 |
| TAM-02 | Bajio | -21.8 | -8.6 | -19.9 | -7.8 | -28.9 | -11.3 | 12.5 | 4.9 |
| TAM-03 | Bajio | -8.9 | -2.8 | 23.9 | 7.6 | 10.8 | 3.4 | 71.3 | 22.5 |
| TAM-06 | Bajio | -28.8 | -8.6 | -2.0 | -0.6 | -14.7 | -4.4 | 43.8 | 13.1 |
| TAM-09 | Bajio | -12.8 | -4.8 | -22.6 | -8.4 | -32.5 | -12.1 | 13.4 | 5.0 |
| TAM-20 | Bajio | -35.0 | -7.7 | -39.1 | -8.5 | -56.0 | -12.2 | 21.8 | 4.8 |
| TAM-21 | Bajio | -0.1 | 0.0 | 114.0 | 20.1 | 86.4 | 15.2 | 213.5 | 37.6 |
| TAM-22 | Bajio | -39.8 | -8.2 | -61.8 | -12.7 | -78.8 | -16.1 | -0.5 | -0.1 |

**Table S3**. Across-Amazonia multiple regression model of aboveground biomass (AGB) as a function of basal area and basal area weighted wood density (cf Fig. 6). Aboveground biomass was log-transformed to meet model assumptions. This model explained 90.0 % of variation in AGB. The proportion of this explained independently by each explanatory variable is shown; a further 26.3 % of variation is only explained when both terms were included in the model (‘shared’ variation). N = 165 forest inventory plots. The remaining 10 % of variation must be due to either variation in height-diameter allometry, or due to the specific configuration of which trees in a plot have high wood density. Adding allometric region into the model (to account for height-diameter allometry) takes explained variation up to 95.9%, suggesting that ≈4 % of variation is due to the configuration of which trees in a plot have high or low wood density.

| Variable | Coefficient | SE | t | P | Explained variation (%) |
| --- | --- | --- | --- | --- | --- |
| Intercept | 3.028 | 0.073 | 41.6 | <0.001 |  |
| Basal area | 0.050 | 0.002 | 26.3 | <0.001 | 42.8 |
| Basal-area weighted wood density | 1.989 | 0.108 | 18.4 | <0.001 | 20.8 |

**Table S4.** As Table S3, but using the abundance weighted mean wood density rather than basal area weighted wood density. This model explained 88.2 % of variation in AGB; 24.2 % of this explanatory power was only present when both basal area and wood density were included in the same model (‘shared’ variation).

| Variable | Coefficient | SE | t | P | Explained variation (%) |
| --- | --- | --- | --- | --- | --- |
| Intercept | 2.898 | 0.087 | 33.3 | <0.001 |  |
| Basal area | 0.051 | 0.002 | 24.9 | <0.001 | 45.0 |
| Wood density | 2.150 | 0.133 | 16.2 | <0.001 | 19.1 |

**Table S5.** Coefficients of the bivariate regression models (Fig. 6 – 8, Fig. S1 and Fig. S2). Variable abbreviations are: AGB (Aboveground biomass), WD (wood density), WD_BA_ (basal area weighted wood density), BA (basal area), AGWP (aboveground wood production), Mort_AGB_ (biomass mortality) and Mort_Stem_ (stem mortality).

|  | *β* | SE | *t* | *P* | *R^2^* |
| --- | --- | --- | --- | --- | --- |
|  | ln(AGB) = α + β WD_BA_ + ε | | | | |
| Pan-Amazon | 2.852 | 0.236 | 12.06 | <0.001 | 0.472 |
| West Amazon | 0.827 | 0.414 | 2.00 | 0.050 | 0.051 |
| Brazilian Shield | 3.237 | 2.221 | 1.46 | 0.179 | 0.191 |
| East-central Amazon | 2.802 | 0.620 | 4.52 | <0.001 | 0.369 |
| Guiana Shield | 3.806 | 0.464 | 8.20 | <0.001 | 0.633 |
|  | ln(AGB) = α + β BA+ ε | | | | |
| Pan-Amazon | 0.060 | 0.003 | 19.11 | <0.001 | 0.691 |
| West Amazon | 0.037 | 0.003 | 12.09 | <0.001 | 0.664 |
| Brazilian Shield | 0.072 | 0.005 | 14.38 | <0.001 | 0.958 |
| East-central Amazon | 0.061 | 0.004 | 14.15 | <0.001 | 0.851 |
| Guiana Shield | 0.050 | 0.003 | 18.61 | <0.001 | 0.899 |
|  | BA = α + β WD_BA_+ ε | | | | |
| Pan-Amazon | 17.301 | 4.271 | 4.05 | <0.001 | 0.091 |
| West Amazon | -22.311 | 9.004 | -2.48 | 0.015 | 0.077 |
| Brazilian Shield | 32.461 | 31.649 | 1.03 | 0.332 | 0.105 |
| East-central Amazon | 27.419 | 10.955 | 2.50 | 0.017 | 0.152 |
| Guiana Shield | 57.641 | 11.091 | 5.20 | <0.001 | 0.409 |
|  | ln(AGWP) = α + β WD_BA_+ ε | | | | |
| Pan-Amazon | -0.339 | 0.247 | -1.37 | 0.172 | 0.011 |
| West Amazon | 0.376 | 0.483 | 0.78 | 0.438 | 0.008 |
| Brazilian Shield | -1.448 | 1.355 | -1.07 | 0.313 | 0.113 |
| East-central Amazon | -1.900 | 0.604 | -3.14 | 0.003 | 0.220 |
| Guiana Shield | -1.121 | 0.772 | -1.45 | 0.154 | 0.051 |
|  | ln(Mort_AGB_) = α + β WD_BA_+ ε | | | | |
| Pan-Amazon | -0.199 | 0.599 | -0.33 | 0.740 | 0.001 |
| West Amazon | 1.438 | 1.147 | 1.25 | 0.214 | 0.021 |
| Brazilian Shield | -1.004 | 1.901 | -0.53 | 0.610 | 0.030 |
| East-central Amazon | 1.496 | 2.162 | 0.69 | 0.493 | 0.014 |
| Guiana Shield | -7.483 | 2.505 | -2.99 | 0.005 | 0.186 |
|  | ln(Mort_stem_) = α + β WD_BA_+ ε | | | | |
| Pan-Amazon | -3.912 | 0.466 | -8.39 | <0.001 | 0.302 |
| West Amazon | -0.482 | 0.847 | -0.57 | 0.571 | 0.004 |
| Brazilian Shield | -2.646 | 2.600 | -1.02 | 0.336 | 0.103 |
| East-central Amazon | -3.343 | 1.429 | -2.34 | 0.025 | 0.135 |
| Guiana Shield | -7.069 | 2.044 | -3.46 | 0.001 | 0.235 |
|  | ln(AGB) = α + β WD+ ε | | | | |
| Pan-Amazon | 3.101 | 0.278 | 11.14 | <0.001 | 0.432 |
| West Amazon | 0.553 | 0.492 | 1.12 | 0.264 | 0.017 |
| Brazilian Shield | 2.650 | 3.358 | 0.79 | 0.450 | 0.065 |
| East-central Amazon | 3.335 | 0.729 | 4.58 | <0.001 | 0.374 |
| Guiana Shield | 4.212 | 0.504 | 8.36 | <0.001 | 0.642 |
|  | BA = α + β WD+ ε | | | | |
| Pan-Amazon | 18.694 | 4.874 | 3.84 | <0.001 | 0.083 |
| West Amazon | -30.994 | 10.322 | -3.00 | 0.004 | 0.109 |
| Brazilian Shield | 29.146 | 46.024 | 0.63 | 0.542 | 0.043 |
| East-central Amazon | 34.668 | 12.766 | 2.72 | 0.010 | 0.174 |
| Guiana Shield | 69.356 | 11.324 | 6.12 | <0.001 | 0.490 |
|  | ln(AGWP) = α + β WD+ ε | | | | |
| Pan-Amazon | -0.444 | 0.280 | -1.58 | 0.115 | 0.015 |
| West Amazon | 0.146 | 0.565 | 0.26 | 0.796 | 0.001 |
| Brazilian Shield | -2.381 | 1.860 | -1.28 | 0.233 | 0.154 |
| East-central Amazon | -1.859 | 0.744 | -2.50 | 0.017 | 0.151 |
| Guiana Shield | -1.194 | 0.850 | -1.40 | 0.168 | 0.048 |
|  | ln(Mort_AGB_) = α + β WD+ ε | | | | |
| Pan-Amazon | -0.483 | 0.680 | -0.71 | 0.479 | 0.003 |
| West Amazon | 0.498 | 1.352 | 0.37 | 0.713 | 0.002 |
| Brazilian Shield | -0.617 | 2.707 | -0.23 | 0.825 | 0.006 |
| East-central Amazon | 1.708 | 2.554 | 0.67 | 0.508 | 0.013 |
| Guiana Shield | -7.704 | 2.792 | -2.76 | 0.009 | 0.163 |
|  | ln(Mort_stem_) = α + β WD+ ε | | | | |
| Pan-Amazon | -4.480 | 0.528 | -8.49 | <0.001 | 0.307 |
| West Amazon | -0.870 | 0.985 | -0.88 | 0.380 | 0.010 |
| Brazilian Shield | -4.292 | 3.587 | -1.20 | 0.262 | 0.137 |
| East-central Amazon | -3.956 | 1.687 | -2.35 | 0.025 | 0.136 |
| Guiana Shield | -7.331 | 2.284 | -3.21 | 0.003 | 0.209 |
|  | ln(Mort_stem_) = α + β ln(AGB)+ ε | | | | |
| Pan-Amazon | -0.988 | 0.110 | 9.00 | <0.001 | 0.332 |
| West Amazon | -0.241 | 0.230 | 1.05 | 0.298 | 0.015 |
| Brazilian Shield | -0.550 | 0.322 | 1.71 | 0.122 | 0.244 |
| East-central Amazon | -0.732 | 0.309 | 2.37 | 0.023 | 0.138 |
| Guiana Shield | -1.679 | 0.408 | 4.12 | <0.001 | 0.303 |
|  | ln(Mort_AGB_) = α + β ln(AGB)+ ε | | | | |
| Pan-Amazon | 0.062 | 0.144 | 0.43 | 0.669 | 0.001 |
| West Amazon | 0.368 | 0.314 | 1.17 | 0.245 | 0.018 |
| Brazilian Shield | 0.130 | 0.257 | 0.51 | 0.625 | 0.028 |
| East-central Amazon | 0.556 | 0.462 | 1.20 | 0.237 | 0.040 |
| Guiana Shield | -1.596 | 0.521 | 3.06 | 0.004 | 0.194 |

**Supplementary Figures**


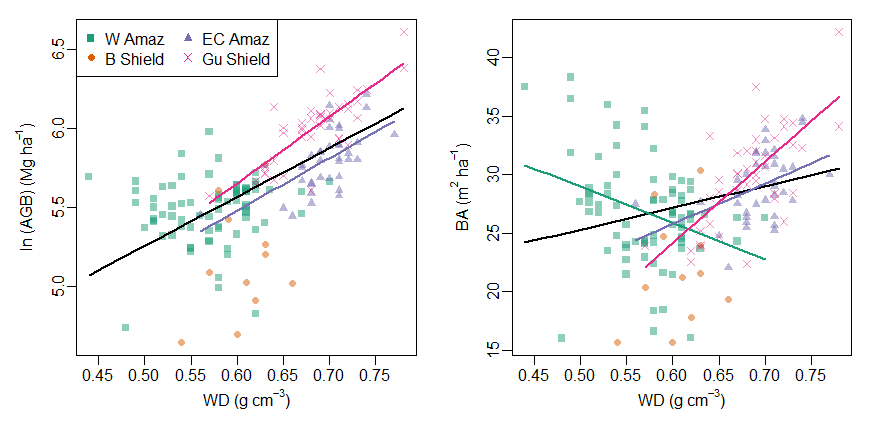
**Figure S1.** As Fig. 6, but using abundance-weighted mean wood density instead of basal-area weighted wood density. Note that wood density and basal area are positively correlated across Amazonia (r = 0.302), but correlations within regions range from negative (r = -0.330, W Amazon) to strongly positive (r = 0.700, Guiana Shield).


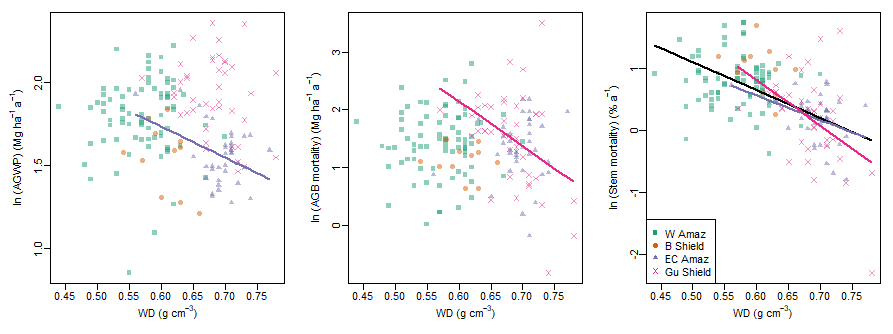
**Figure S2.** As Fig. 7, but using abundance-weighted mean wood density instead of basal-area weighted wood density.
